# The Photodegradation of Quercetin: Relation to Oxidation

**DOI:** 10.3390/molecules17088898

**Published:** 2012-07-26

**Authors:** Stefano Dall’Acqua, Giorgia Miolo, Gabbriella Innocenti, Sergio Caffieri

**Affiliations:** Department of Pharmaceutical and Pharmacological Sciences, University of Padova, Via F. Marzolo 5, 35131 Padova, Italy; Email: stefano.dallacqua@unipd.it (S.D.); giorgia.miolo@unipd.it (G.M.); sergio.caffieri@unipd.it (S.C.)

**Keywords:** quercetin, photostability, photodegradation

## Abstract

The photostability of quercetin in alcoholic solutions was studied. Both UVA and UVB light induced degradation of quercetin, yielding a single product **1** deriving from oxidation and addition of an alcohol molecule to the 2,3 double bond. The same mechanism operated when quercetin was dissolved in alkaline solutions, and again a product **2** due to oxidation and addition of water was characterized. Comparison with quercetin analogs confirmed that, despite the presence of five hydroxy groups in quercetin, those in positions 3, 3′, and 4′ are mainly involved in the antioxidant activity of the compound , as well as in its photolability.

## 1. Introduction

**Figure 1:**
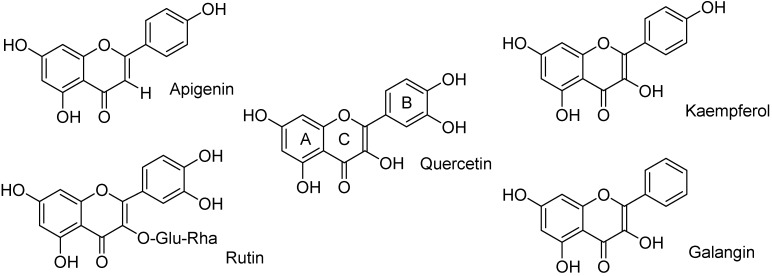
Structures of the studiedflavonoids.

Quercetin (3,5,7,3′,4′-pentahydroxyflavone, Figure 1) is one of the most widespread compounds of the natural flavonoids class. Such compounds are known for their ultraviolet (UV) B photoprotective properties in plants. Under stressful conditions, in different cell compartments and tissue structures, plants accumulate pigments, such as flavonoids, which can attenuate radiation in the UV and visible parts of the spectrum [1]. The mechanism mainly involves the capacity of flavonoids to scavenge free radicals and reactive oxygen species. Due to the presence of many phenolic hydroxy groups, flavonoids can donate hydrogen atoms to radicals, thus acting as radical chain terminators [2]. Newly formed flavonoid radicals are delocalized on the whole molecule, making them far less aggressive. Radical scavenging is probably involved in the beneficial effects exerted by flavonoids in humans, where they have been proven to counteract aging, inflammation, atherosclerosis, and cancer [3].

According to their molecular structure, flavonoids are classified into various groups, all sharing the polyphenolic moiety and antioxidant activity to a greater or lesser extent. However, quercetin possesses a carbonyl group in position 4, a double bond between carbons 2 and 3, and five hydroxy groups in positions 3,5,7,3′,4 (see Figure 1); in particular the three hydroxyl groups in positions 3, 3′, and 4′, make it one of the most potent of the congeners [4].

As flavonoids probably behave in plants as photoprotectors, besides the systemic actions exploited by flavonoids ingested with vegetables or food supplements, it has been suggested that they can be used as sunscreens [5]. To be efficient in its photoprotective action, a sunscreen must have an absorption spectrum wide enough to cover as many of the wavelengths of the UV region as possible, and must be stable under prolonged exposure to UV light. The former requirement is fulfilled by most flavonoids. As regards the latter aspect, despite the very high number of publications dealing with the photoprotective effects of quercetin, the literature reports only a few studies concerning the effect of light on quercetin, and on flavonoids in general, at the molecular level and some of the reports are contradictory. On one hand, quercetin has proved to be photostable in propylene glycol solutions [6]; on the other, its photodegradation has been observed in creams, unless protected in lipid micelles [7] or on cellulose [8]. Kaempferol—which differs from quercetin only in the absence of the 3′-OH—has been shown to be photodegradable [9], although no indications have been given regarding the possible mechanism and resulting products. Other studies have evaluated in detail the degradation of quercetin in the dark, when dissolved in an alkaline medium [10], treated with radical generators [11,12], or electrochemically oxidized [12,13].

The aim of this work was thus to reconsider the photostability of quercetin and some analogs (Figure 1) in solution, to identify its possible photoproducts and to compare them with the products formed in an alkaline medium.

## 2. Results and Discussion

### 2.1. Results

**Figure 2:**
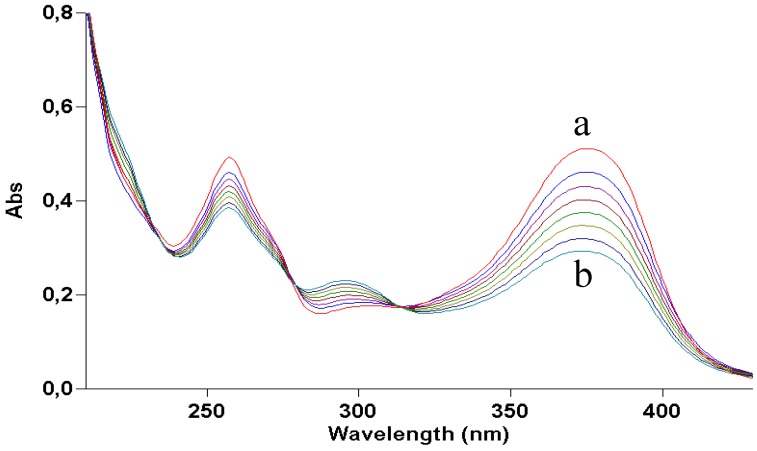
UV absorption spectra of a 2 × 10^−5^ M ethanol solution of quercetin irradiated with increasing doses of UVA. Curve a, dark; curve b, 70 J/cm^2^.

**Figure 3:**
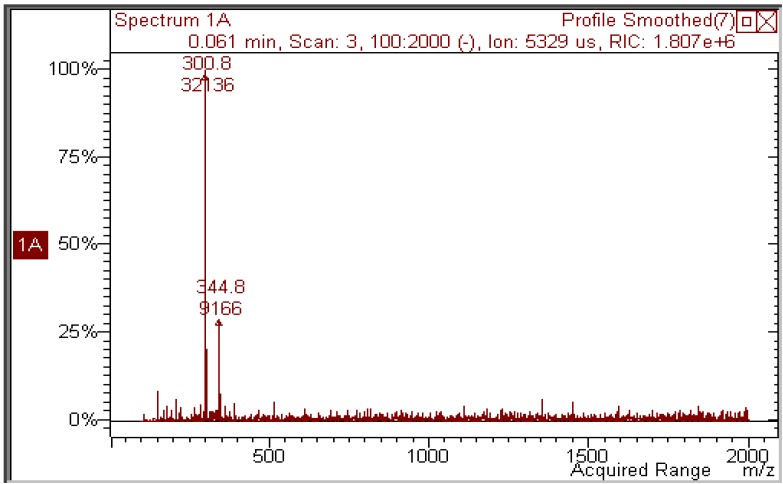
Negative-ion mass spectrum of a 2 × 10^−5^ M solution of quercetin in ethanol irradiated with 50 J/cm^2^ UVA.

**Scheme 1:**
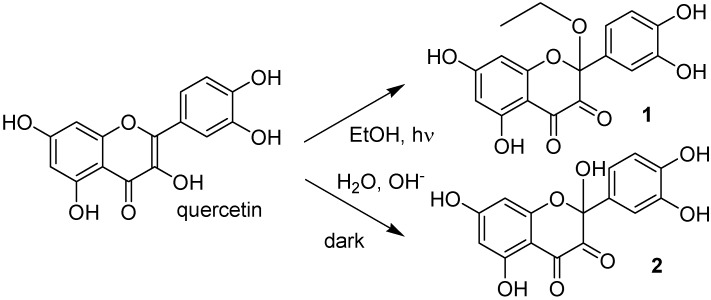
Proposed structure of quercetin products obtained by either UVA irradiation in ethanol (**1**) or in aqueous ammonia in the dark (**2**).

Quercetin was dissolved (2 × 10^−5^ M) in ethanol and irradiated with increasing doses of UVA. Figure 2 shows how irradiation affects the UV absorption spectrum: the maximum at 375 nm undergoes a gradual blue shift to 295 nm, indicating loss of conjugation of the chromophore. The presence of at least two isosbestic points at 275 and 325 nm suggests that at least one or more photoproducts form upon UV light. During irradiation, the solution was also submitted to negative-ion MS analysis. Figure 3 shows that, in the MS spectrum of a sample irradiated with 50 J/cm^2^, only two important peaks appear: the first (*m/z* 300.8) corresponds to the molecular ion of quercetin [M−H]^−^ and the second has *m/z* 344.8, corresponding to a molecular weight of 346 Da. The increase in mw of 44 Da corresponds to the addition of an ethanol molecule to the 2,3 double bond of quercetin (thus explaining the blue shift in the UV absorption spectrum), accompanied by oxidative loss of two hydrogen atoms (compound **1** in Scheme 1). This modification was substantiated by further experiments: (1) removal of oxygen from the solution through bubbling with argon for 10 min before and during irradiation completely abolished photolysis, as did the addition of a small amount of a concentrated aqueous solution of ascorbic acid; (2) when methanol was used, the mw of the photoproduct was 332 (quercetin + MeOH − 2H), but when deuterated methanol was added, the mw was 335, showing that the −OCD_3_ moiety was present in the product; the fourth deuterium atom was lost in the oxidative step. In addition, the proposed structure corresponds to that already obtained by Zhou *et al.* [13] by electrochemical oxidation of quercetin in ethanol.

Experiments were repeated with UVB light, but no significant differences were found in either products or rate of photodegradation. In a non-nucleophilic solvent like acetonitrile, quercetin was stable. Attempts were made to remove the solvent in order to perform NMR analysis on the photoproduct. Unfortunately, even mild conditions like lyophilization were unsuccessful, because of photoproduct instability.

**Figure 4:**
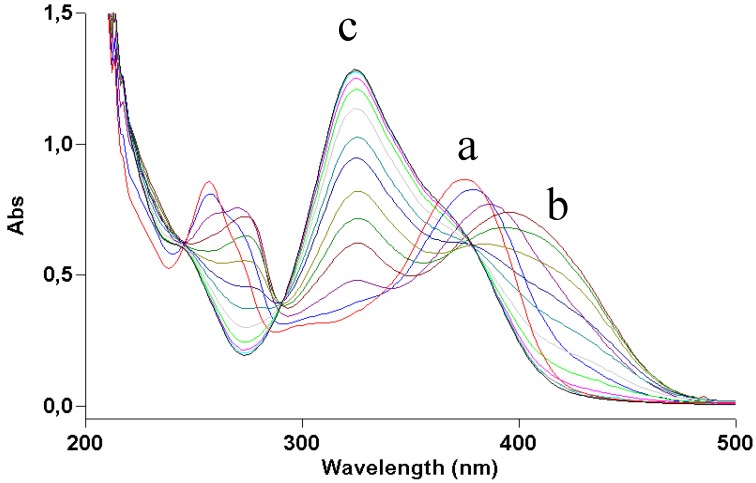
Time-course of UV absorption spectrum of a solution of quercetin in aqueous ammonia. Curve a was recorded immediately after addition of ammonia, curve b after 15 s, curve c after 60 s.

Quercetin is practically insoluble in water. To study its photostability in an aqueous environment, a water suspension of the compound was made slightly alkaline (pH ≈ 9) by adding ammonia. However, as already reported [10], this treatment leads to degradation of the molecule in the dark. Recording fast UV absorption spectra at 5-s intervals gave the results shown in Figure 4. During the first few seconds the spectrum showed a red shift due to ionization of the molecule, quickly followed by a blue shift. The final profile closely resembled that of quercetin irradiated in ethanol (Figure 2), suggesting a similar structure. In this case too, careful removal of oxygen or addition of ascorbic acid stopped the reaction at the ionization step (UV absorption maximum at 395 nm). 

As observed by Zenkevich *et al.* [10] and with the photoproduct formed in ethanol (see above), the product was thermally unstable, making its isolation impossible. However, MS measurements on the crude solution revealed that a single photoproduct was formed, with a mw of 318 Da (quercetin + H_2_O − 2H). When deuterium oxide was used as the solvent, the mw was 319, allowing the product to be assigned to structure **2** (Scheme 1).

The latter experiment—that is, alkalinization of a suspension of quercetin in deuterium oxide—was also possible with higher amounts of quercetin, suitable for ^13^C-NMR experiments: Table 1 compares the spectroscopic data of the product with those of parent quercetin. Only two carbon atoms differ significantly in their resonance between quercetin and the product, *i.e.*, C-2 and C-3, matching the proposed structure. Instead, the ^1^H-NMR spectrum showed that the chemical shift of the non-OH protons, H-6, 7, 2′, 5′, and 6′, was not significantly changed.

**Table 1:** ^13^C-NMR spectroscopic data (75 MHz) for quercetin and product **2**.

Position	Quercetin (in CD_3_OD)	Product 2 (in D_2_O + NH_3_)
2, qC	147.5	113.7
3, qC	136.5	180.2
4, qC	176.5	174.4
5, qC	161.0	157.9
6, CH	99.5	101.7
7, qC	166.0	159.8
8, CH	94.5	96.3
9, qC	156.7	154.5
10, qC	104.0	102.0
1′, qC	123.0	121.6
2′, CH	116.0	116.9
3′, qC	145.7	141.9
4′, qC	148.1	149.3
5′, CH	116.5	117.2
6′, CH	121.0	120.3

Both photolysis of quercetin in alcohols and degradation in alkalis thus proceed through two steps: (i) addition of solvent; and (ii) oxidation (not necessarily in this order, see Discussion).

Further support to the similarity of compounds **1** and **2** came from MS/MS experiments: in both, the first fragmentation was the loss of the group in position 3 (OEt or OH, respectively) followed by the same fragmentation pattern.

For oxidation, the presence of a hydroxy group in position 3 seems to be mandatory. Four flavonoid analogs of quercetin were then studied (Figure 1). In apigenin, the hydroxy group was substituted by a hydrogen atom; in rutin it was glycosylated. Both compounds, dissolved in methanol, proved to be stable under UV irradiation. When dissolved in aqueous ammonia, both underwent deprotonation—with consequent redshift of the UV absorption spectrum—but no further degradation occurred.

**Figure 5:**
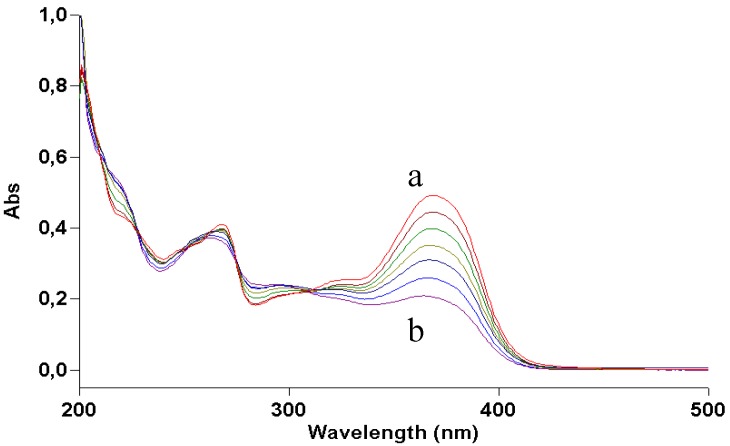
UV absorption spectra of a 2 × 10^−5^ M methanol solution of kaempferol irradiated with increasing doses of UVA. Curve a, dark; curve b, 70 J/cm^2^.

**Figure 6:**
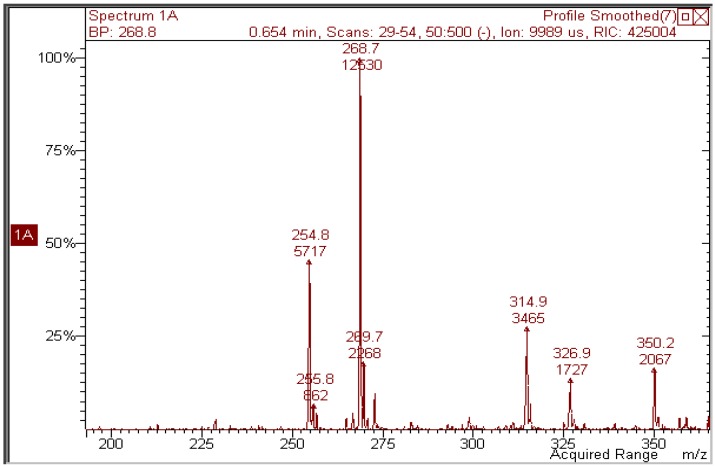
Negative-ion mass spectrum of a 2 × 10^−5^ M methanol solution of kaempferol irradiated with 70 J/cm^2^ UVA.

Kaempferol and galangin (Figure 1), which lack one or both hydroxy groups of ring B while maintaining the hydroxy group in 3, were expected to behave like quercetin. This was only partly true for the former (Figure 5): comparison of its photolysis in methanol with that of quercetin (Figure 2) shows a similar decrease in absorption at 380 nm, whereas both decrease at 280 and increase at 300 are less definite, like the isosbestic points. The mass spectrum (Figure 6) does show that the peak of the expected photoproduct at *m/z* 314.9 is a minor signal with respect to other unidentified ones.

**Figure 7:**
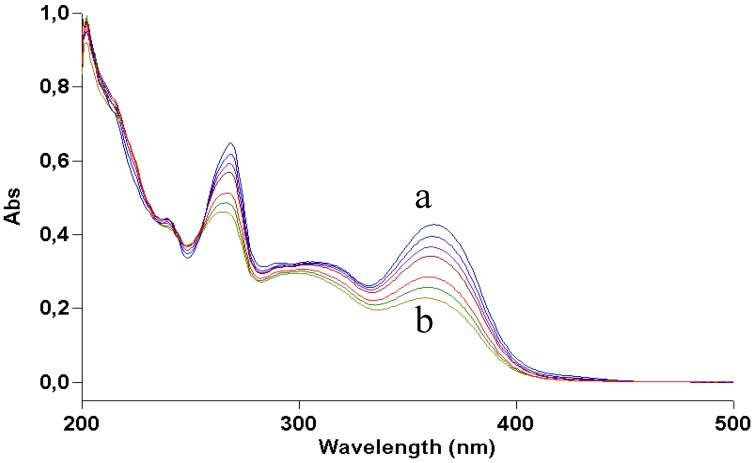
UV absorption spectra of a 2 × 10^−5^ M methanol solution of galangin irradiated with increasing doses of UVA. Curve a, dark; curve b, 70 J/cm^2^.

**Figure 8:**
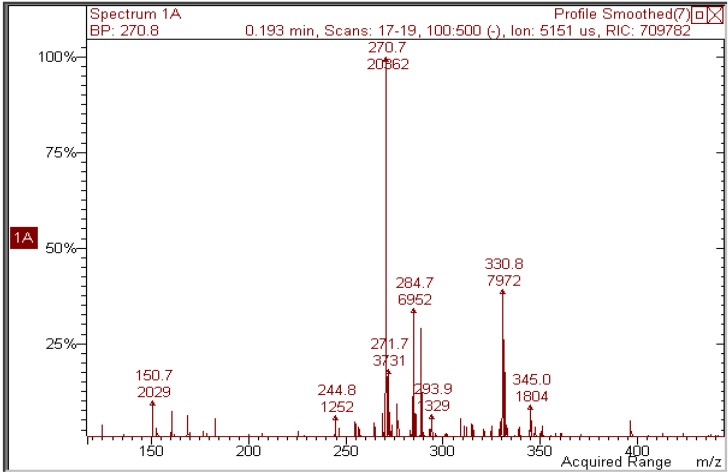
Negative-ion mass spectrum of a 2 × 10^−5^ M methanol solution of galangin irradiated with 70 J/cm^2^ UVA.

Results obtained by dissolving kaempferol and galangin in aqueous ammonia were consistent with the irradiation experiments. In the former, the product deriving from water addition and oxidation was present, although mixed with several byproducts; the latter was only deprotonated without further degradation (Figure 7 and Figure 8).

### 2.2. Discussion

The photostability of quercetin in nucleophilic solvents (MeOH and EtOH) was studied. One photoproduct (**1** in Scheme 1) was characterized by mass spectrometry as coming from addition of the alcohol to the 2,3 double bond of the flavone and oxidation by air, but proved unstable to isolation procedures. Addition may precede oxidation or *vice versa*. On the one hand, in the former hypothesis the structure of the possible quercetin-ethanol adduct closely resembles that of ascorbic acid: if addition occurs first, oxidation should then be easy. On the other hand, studying the cooperation between quercetin and ascorbate on the suppression of photosensitized hemolysis, Sorata *et al.* [14] found that ascorbate reduced the oxidized form of quercetin, rather than acting as a singlet oxygen and radical quencher. There are other examples of photoaddition of nucleophiles to the vinyl bond of β-unsaturated ketones. To mention only one, the 5,6 double bond of the antitumor agent 5-fluorouracil adds water on irradiation [15]. However, rutin, which has a non-oxidizable electron-drawing group in position 3, like 5-fluorouracil, is photostable, thus ruling out the possibility that addition is the first step of the process.

As regards the behaviour of the flavones in alkaline medium, Zenkevich *et al.* [10] were not able to characterize the primary photoproduct because of its instability. On the basis of the final degradation products (floroglucinol, 3,4-dihydroxybenzoic acid, and 2,4,6-trihydroxybenzoic acid) they proposed an intermediate of depsidic nature, in which ring C is open. In the present paper, by means of MS and ^13^C-NMR, the structure of the primary product (compound **2** in Scheme 1) was shown to be similar to that obtained with light: it derives from oxidation and the addition of water. The depside is probably formed from **2** in a later step, during the removal of the solvent, as the final products mentioned above were also found in this study.

Kaempferol and galangin have lower antioxidant properties than quercetin [16]. In fact, both under irradiation in methanol and in the dark in alkaline medium, their behaviour is different from that of quercetin: slightly different when one hydroxy group still remains on ring C (kaempferol), and drastically, when both hydroxy groups are removed (galangin).

As Figure 2 shows, the light dose necessary to destroy quercetin is very high: about 70 J/cm^2^ for 50% degradation. The quantum yield of photodegradation was roughly estimated to be of the order of 10^−4^. The percentage of modified molecules therefore becomes negligible in more concentrated solution. This fact may explain the stability of quercetin observed by Choquenet [5], as it was measured at a concentration as high as 10% (w/w), necessary to achieve a suitable sun protection factor. Thus, the photodegradation of quercetin should not be a problem in such conditions.

## 3. Experimental

### General

Quercetin, apigenin, galangin, kaempferol, and rutin were from Sigma-Aldrich (Milan, Italy). NMR-grade D_2_O and CD_3_OD were from E. Merck (Darmstadt, Germany). Irradiations were performed in quartz cuvettes (1 × 1 cm; total volume 3 mL) with Philips HPW125 lamps (emitting mainly at 365 nm) or Philips PL-S 9W/12 lamps (emitting in the UVB region, 290–330 nm, peak at 312 nm). The distance between the lamps and the cuvette was of 5 cm. The total energy striking the sample was monitored on a radiometer (Mod. 97503, Cole-Parmer Instrument Company, Niles, IL, USA), equipped with a 365-CX or a 312-CX sensor. Radiation intensity was 0.30 and 0.20 J/cm^2^ min for UVA and UVB, respectively. Irradiation times were based on the total UVA/UVB intensity given to the samples (50–70 J/cm^2^). The temperature was maintained at about 25 °C in a thermostated chamber by means of an air circulating flow. UV absorption spectra were recorded on a Cary 50 spectrophotometer (Varian, Leiny, Italy). NMR spectra were recorded on a AMX 300 spectrometer (Bruker Biospin, Milan, Italy), operating at 300 MHz for ^1^H and 75 MHz for ^13^C. MS measurements were performed on a Varian 500 MS ion trap mass spectrometer equipped with an electrospray ion source. Samples were injected into the ESI source via a syringe pump at a flow rate of 10 μL/minute. MS parameters were as follows: nebulizer gas pressure 25 psi, drying gas temperature 320 °C, drying gas pressure 15 psi, needle voltage −4,500 V, spray shield voltage −600 V, RF loading 75%, capillary voltage 100 V, 3.25 s/scan. Multiple MS experiments were performed by isolating either [M−H]^−^ or fragment ions and applying appropriate values of excitation amplitude, excitation times and excitation storage levels (calculated by MS software on the basis of the selected ion).

## 4. Conclusions

The photostability of quercetin in nucleophilic solvents was studied. A single photoproduct **1** was formed deriving from addition of a solvent molecule to the 2,3 double bond of quercetin and oxidation by air. The same mechanism operated when quercetin was dissolved in alkaline solutions, and again a product **2** due to oxidation and addition of water was characterized. In non nucleophilic solvent the quercetin was stable. The comparison with quercetin analogs confirmed that the hydroxy groups in positions 3, 3′, and 4′ are involved not only in the antioxidant activity of quercetin, but also in its photolability. Therefore, the OH groups in positions 5 and 7 do not play a crucial role in the phototoxidative mechanism.
